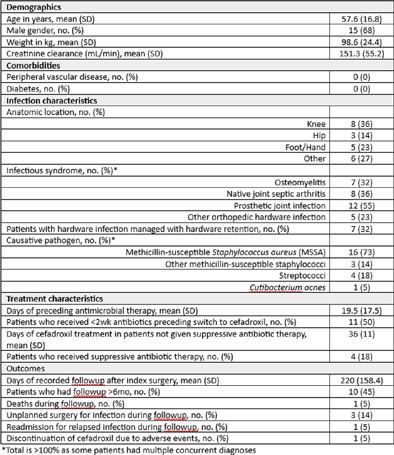# Cefadroxil is an option for Gram-positive bone and joint infections in adults

**DOI:** 10.1017/ash.2024.143

**Published:** 2024-09-16

**Authors:** Kennedy Kluthe, Rachael Johnson, Nicolas Cortes-Penfield

**Affiliations:** Student; Nebraska Medicine; University of Nebraska Medical Center

## Abstract

**Background:** Oral antimicrobial therapy for osteoarticular infections in adults is increasingly adopted; however, the preponderance of published data and experience with oral antibiotics involves agents such as fluoroquinolones, trimethoprim-sulfamethoxazole, and clindamycin. Data supporting the use of narrower-spectrum, stewardship-friendly oral antibiotics with favorable toxicities profiles (e.g. cefadroxil) are needed. **Methods:** We report a case series of adults who underwent surgery for monomicrobial Gram-positive osteomyelitis, native joint septic arthritis (NJSA), prosthetic joint infection (PJI), or other orthopedic hardware infection at our institution from January 2019 through January 2022 and who subsequently received at least two weeks of oral cefadroxil. We excluded patients who had polymicrobial infection or curative amputation, or who received cefadroxil only as suppressive antibiotic therapy (SAT). Our primary outcome of interest was treatment success during followup, defined as freedom from death, unplanned surgery for infection, or readmission for worsening infection. **Results:** We identified 22 patients who received cefadroxil for primary treatment of osteoarticular infection. The mean age was 57.6 years, mean weight 98.6 kg, 68% were male, and none had peripheral vascular disease. Infections included PJI in 12, NJSA in 8, osteomyelitis in 7, and other hardware infections in 5; some patients had multiple infectious syndromes. Methicillin-susceptible Staphylococcus aureus (MSSA) was the most common pathogen (73%), followed by streptococci (18%), other methicillin-susceptible staphylococci (14%), and Cutibacterium acnes (5%). Half of patients received 120kg who had device infections with MSSA, two of which were managed with implant retention, suggesting pharmacokinetic/pharmacodynamic factors and biofilm burden could influence treatment outcome. Limitations of this study include its small sample size, noncomparative nature, lengthy initial intravenous antibiotic durations, and limited durations of followup. These data suggest cefadroxil merits investigation for adult osteoarticular infection in larger comparative (and ideally, prospective randomized) studies.

**Figure:**